# Efficacy of Carbazole Alkaloids, Essential Oil and Extract of *Murraya koenigii* in Enhancing Subcutaneous Wound Healing in Rats

**DOI:** 10.3390/molecules171214449

**Published:** 2012-12-05

**Authors:** Thilahgavani Nagappan, Thirukanthan Chandra Segaran, Mohd Effendy Abdul Wahid, Perumal Ramasamy, Charles S. Vairappan

**Affiliations:** 1 Laboratory of Natural Products Chemistry, Institute for Tropical Biology and Conservation, Universiti Malaysia Sabah, 88999 Kota Kinabalu, Sabah, Malaysia; E-Mail: thila_vani@yahoo.com.my; 2 Institute of Marine Biotechnology, University Malaysia Terengganu, 21030 Kuala Terengganu, Terengganu, Malaysia; E-Mails: thiru@umt.edu.my (T.C.S.); effendy@umt.edu.my (M.E.A.W.); 3 School of Medicine, Universiti Malaysia Sabah, 88999 Kota Kinabalu, Sabah, Malaysia; E-Mail: peruram@hotmail.com

**Keywords:** *Murraya koenigii*, extract, mahanimbicine, wound healing, collagen deposition

## Abstract

The traditional use of *Murraya koenigii* as Asian folk medicine prompted us to investigate its wound healing ability. Three carbazole alkaloids (mahanine (**1**), mahanimbicine (**2**), mahanimbine (**3**)), essential oil and ethanol extract of *Murraya koenigii* were investigated for their efficacy in healing subcutaneous wounds. Topical application of the three alkaloids, essential oil and crude extract on 8 mm wounds created on the dorsal skin of rats was monitored for 18 days. Wound contraction rate and epithelialization duration were calculated, while wound granulation and collagen deposition were evaluated *via* histological method. Wound contraction rates were obvious by day 4 for the group treated with extract (19.25%) and the group treated with mahanimbicine (**2**) (12.60%), while complete epithelialization was achieved on day 18 for all treatment groups. Wounds treated with mahanimbicine (**2**) (88.54%) and extract of *M. koenigii* (91.78%) showed the highest rate of collagen deposition with well-organized collagen bands, formation of fibroblasts, hair follicle buds and with reduced inflammatory cells compared to wounds treated with mahanine (**1**), mahanimbine (**3**) and essential oil. The study revealed the potential of mahanimbicine (**2**) and crude extract of *M. koenigii* in facilitation and acceleration of wound healing.

## 1. Introduction

In most Asian countries, herbal products play an important role in the treatment of wounds, burns, intestinal problems, coughs and general torpor [1]. Use of traditional remedies and plants in the treatment of burns and wounds is an important aspect of health management and is also an efficient way to promote cheaper healthcare options [2,3]. Removal and prevention of infection is essential for rapid and effective wound healing. Many researchers have reported *in vitro* and *in vivo* evidence to support the use of various plant materials as topical anti-microbial agents to enhance wound healing [4,5,6]. Several indigenous plants and formulations for the management of cuts, bruises, burn and wounds have been described in folkloric as well as the Ayurvedic system of medicine [7,8].

Wound healing is a process of removing damaged tissues or invaded pathogens from the body to restore the continuity and architecture of cutaneous and/or visceral defects [9]. This complex cascade of events starts from the moment of injury and continues for varying periods of time. The process can be categorized into three distinct stages: inflammatory phase (establishment of homeostasis and inflammation), proliferative phase (granulation, contraction and epithelialization time) and remodeling phase, which will determine the ultimate strength and appearance of the healed tissues [3].

Despite the advances of modern medicine in disease management and healing, more than 80% of the world’s population still depends on traditional medicines for various skin diseases inclusive of wound healing [10]. Only 1%–3% of the drugs listed in the western pharmacopoeias are intended for the use as topical remedies, whereas approximately one-third of all traditional medicines can be used for wounds and skin disorders [9,10,11].

On the other hand, research on wound healing agents is a developing area in modern biomedical science [7]. Inflammation is a complex biological response of vascular tissues towards harmful stimuli such as pathogens, damaged cells or irritants. It is a well-structured defense mechanism to remove the injurious stimuli and initiate tissue healing. However, inflamed tissues can respond to various stimuli *via* different bioactive mediators, which at time could amplify the phlogistic reaction due to the interaction between cell types and molecules. In current clinical practice, administration of steroidal and non-steroidal anti-inflammatory drugs is common. Despite being known for their efficacy, a number of undesirable side effects have also been documented [12].

As such, the use of *Murraya koenigii* could be a possible candidate in wound healing since it is widely used in traditional medicine. Well known for its aromatics, *M. koenigii* is consumed in many Asian cuisines. The leaves had been reported to possess pharmacological activities as stimulants, tonics, and as carminative agents to treat influenza, fever, bronchial asthma and animal bites. In addition, the carbazole alkaloids isolated from *M. koenigii* has been reported to induce apoptosis in human leukemia cells, prostate cancer cells and histiocyctic lymphoma cells [13,14,15,16,17,18,19,20]. To further explore the pharmacological potential of this plant, the present study was carried out to investigate the wound healing potential of carbazole alkaloids, essential oil and crude extract of *M. koenigii*.

## 2. Results and Discussion

### 2.1. Chemistry

#### 2.1.1. Identification and Structure Elucidation of Carbazole Alkaloids

**Figure 1:**
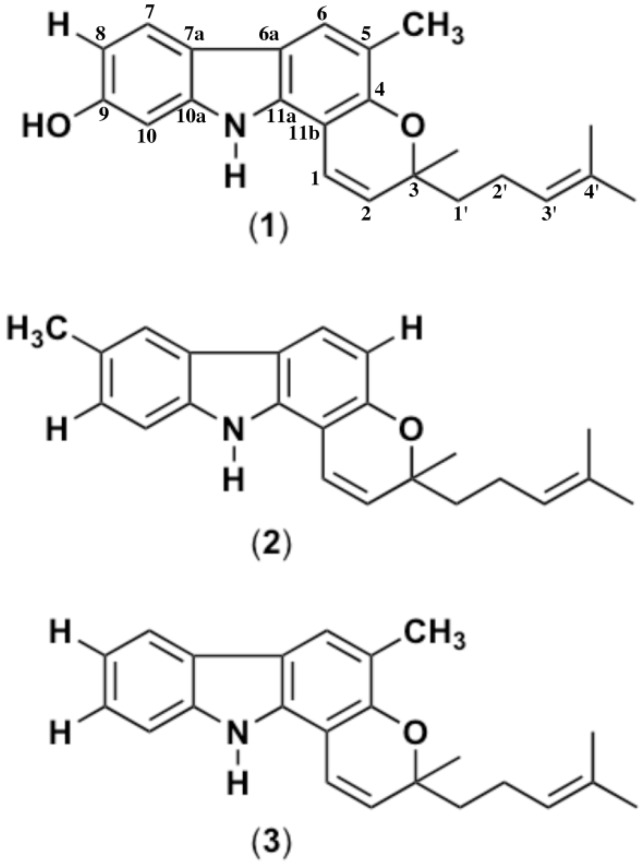
Chemical structures of carbazole alkaloids (mahanine (**1**), mahanimbicine (**2**), mahanimbine (**3**)) isolated from leaves of *Murraya koenigii* (L.) Spreng.

A total of 270 g of fresh leaves were extracted in ethanol and gave 15.6 g of dark green ethanol. Secondary metabolites were isolated and purified via silica gel flush chromatography in solvent gradient and successive high performance liquid chromatography (HPLC) separation as described in the Experimental section. The isolated compounds were subjected to 1D, 2D and other spectroscopic measurements. Based on independent structure elucidation the structures of these three compounds were determined as mahanine (**1**) (0.40%) (C_23_H_25_NO_2_), mahanimbicine (**2**) (0.24%) (C_23_H_25_NO) and mahanimbine (**3**) (0.66%) (C_23_H_25_NO) (Figure 1). Spectroscopic data obtained from this study were similar with the one reported by Ramsewak *et al.* (1999), Tachibana *et al.*, (2001) and Rahman *et al.*, (2005).

#### 2.1.2. Profiling of the Essential Oil

A total of 0.12% (w/w) of aromatic yellow oil was obtained from leaves of *M. koenigii* by hydro distillation and 1 μL was subjected to Gas Chromatography-Mass Spectrometry (GC-MS) analysis. The volatile aromatic hydrocarbons were identified based on their Retention Indices (AART) and mass fragmentation patterns with reference to the NIST 08 and FFNSC version 1.2 databases. A total of 34 aromatic volatile constituents were identified from the oil of *M. koenigii*, where two sesquiterpene hydrocarbons, β-caryophyllene (19.50%) and α-humulene (15.24%), were present as the major volatile metabolites. Detailed analysis revealed that the 34 volatile constituents could be further categorized into twelve oxygenated monoterpenes, twelve sesquiterpene hydrocarbons, nine oxygenated sesquiterpenes and one oxygenated diterpene. The identified volatile constituents from the oil of *M. koenigii* investigated are presented in Table 1.

**Table 1:** Composition (%) of volatile compounds in essential oil of *Murraya koenigii* from Sabah, Malaysia.

RT (min)	Ref RI	RI	Volatile Compound	Concentration (%)
15.73	1082 ^a^	1079	Linalol	0.56
15.91	1099 ^b^	1095	*trans*-Sabinene hydrate	0.53
17.01	1109 ^a^	1112	*trans*-2-Cyclohexen-1-ol	0.48
17.88	1110 ^a^	1113	*cis*-2-Cyclohexen-1-ol	0.54
19.70	1189 ^b^	1185	*para*-Cymen-8-ol	10.31
20.42	1143 ^b^	1139	β-Terpineol	2.52
21.03	1175 ^a^	1170	*trans*-Piperitol	0.40
21.74	1276 ^a^	1273	Chrysanthenyl acetate	0.39
24.16	1284 ^b^	1279	Lavandulyl acetate	1.67
24.37	1285 ^b^	1285	Bornyl acetate	1.68
28.31	1375 ^b^	1370	α-Copaene	0.82
28.91	1390 ^b^	1385	β-Elemene	0.35
29.39	1394 ^a^	1390	(*Z*)-Jasmone	0.11
30.29	1494 ^a^	1489	β-Caryophyllene	19.50
31.09	1438 ^b^	1436	Aromadendrene	0.72
31.84	1454 ^b^	1448	α-Humulene	15.24
32.70	1420 ^a^	1425	Butanedioic acid	2.18
33.29	1487 ^b^	1480	β-Selinene	3.81
33.30	1470 ^a^	1472	Naphthalene	1.90
33.55	1474 ^a^	1478	α-Selinene	6.10
34.37	1518 ^b^	1512	δ-Cadinene	2.03
36.03	1562 ^b^	1566	Nerolidol	2.64
36.05	1564 ^b^	1569	*trans*-Nerolidol	1.32
36.28	1475 ^a^	1481	Cycloheptane	0.13
36.92	1576 ^b^	1580	Spathulenol	1.98
37.13	1587 ^b^	1591	Caryophyllene oxide	2.14
37.26	1594 ^b^	1590	Viridiflorol	1.51
38.13	1598 ^a^	1592	2-Naphthalenemethanol	0.66
38.26	1079 ^b^	1074	Trivertal	0.35
38.55	1696 ^b^	1694	Juniper camphor	1.57
38.83	1581 ^b^	1579	Cubenol	0.57
39.44	1472 ^a^	1476	β-Cadina-1(6),4-diene	0.50
40.16	1593 ^a^	1596	Selina-6-en-4-ol	4.78
54.95	2106 ^b^	2105	Phytol	10.07
**Composition of grouped volatile compounds (%)**				
*Monoterpenes (oxygenated)*				35.29
*Sesquiterpenes (hydrocarbon)*				35.29
*Sesquiterpenes (oxygenated)*				26.47
*Diterpenes (oxygenated)*				2.94

* Identification of volatile components is based on mass spectra value in reference to NIST 08 (^a^) and FFNSC Ver.1.2 (^b^) standard libraries.

### 2.2. Biology

#### 2.2.1. Wound Contraction

**Figure 2:**
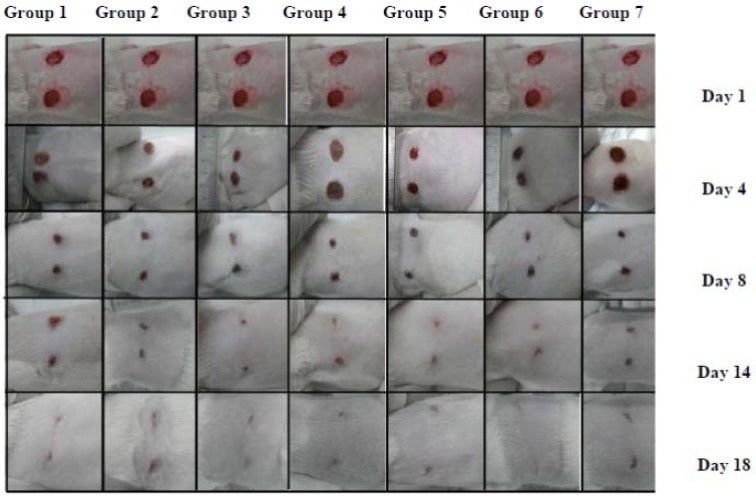
Evidence of wound contraction for animals treated with carbazole alkaloids (mahanine (G2), mahanimbicine (G3), mahanimbine (G4)), essential oil (G5) and extract of *Murraya koenigii* (G6) along with wound treated with standard ointment (G7) and untreated wound (G1).

Wound contraction rates were measured on a daily basis until the wound closure on the wound bed was completely re-epithelialized and filled with new tissue. In all groups investigated, wound contraction rates increased with time. Compared to the rest of the experimental groups, on 4th day post wounding, the wound contraction for animals treated with extract reached 19.25%, followed by wound contraction for animals treated with mahanimbicine (**2**, 12.60%). From the fifth day onwards, the formation of scabs was observed on the treated wounds. On the 14th day post wounding, almost all the wounds under treatment had achieved more than 50.00% of wound contraction. Wounds treated with extract achieved complete epithelialization on the 16th day, while the rest of wounds achieved complete epithelialization by the 18th day (Figure 2, Table 2).

**Table 2:** Effect of carbazole alkaloids [mahanine (G2), mahanimbicine (G3), mahanimbine (G4)], essential oil (G5) and extract (G6) of *Murraya koenigii*, normal healing (G1) and standard ointment (G7) on percentage of wound healing and epithelialization period of excision wound model in rats.

Group	Post-wounding days										Epithelialization period (days)
	2	4	6	8	10	12	14	16	17	18	
1	2.12 ± 4.12	6.25 ± 4.12	10.6 ± 2.93	15.9 ± 3.66	25.8 ± 4.09	40 ± 2.26	57.5 ± 1.78	82.7 ± 2.13	90 ± 1.22	100	18
2	4.6 ± 3.77	8.6 ± 4.04	10.7 ± 3.16	18.0 ± 3.21	35 ± 2.34	45.5 ± 2.11	62 ± 1.08	88.5 ± 2.03	96 ± 1.35	100	18
3	6.25 ± 2.42	12.6 ± 3.92	22.0 ± 3.12	27.0 ± 3.24	38 ± 4.05	52.5 ± 1.09	68.5 ± 1.87	90.7 ± 2.88	98 ± 1.30	100	18
4	6.5 ± 3.72	6.5 ± 3.88	12.5 ± 4.86	20 ± 3.56	39.5 ± 3.75	50.5 ± 0.98	65 ± 1.88	93 ± 2.04	97.2 ± 1.05	100	18
5	5.5 ± 3.03	7.5 ± 4.13	10.7 ± 5.67	17.5 ± 2.61	36 ± 4.23	46 ± 2.57	63 ± 1.09	92.5 ± 2.45	95.5 ± 0.84	100	18
6	6.25 ± 2.42	19.25 ± 3.12	37.9 ± 3.09	49.5 ± 1.09	67.5 ± 1.01	75 ± 0.78	95.5 ± 0.66	100 ± 0.55	-	-	16
7	3.5 ± 3.11	7.7 ± 2.02	17.9 ± 2.08	20.5 ± 3.34	37.8 ± 3.12	45 ± 2.11	61 ± 0.76	90 ± 0.84	95 ± 0.83	100	18

*n* = 3 female Sprague-Dawley rats per group, tabular value represent mean ± S.D, *p* ≤ 0.05; Group 1: wound without treatment; Group 2: wound treated with mahanine (**1**); Group 3: wound treated with mahanimbicine (**2**); Group 4: wound treated with mahanimbine (**3**) Group 5: wound treated with essential oil; Group 6: wound treated with extract; Group 7: wound treated with standard wound healing ointment.

#### 2.2.2. Collagen Density

**Figure 3:**
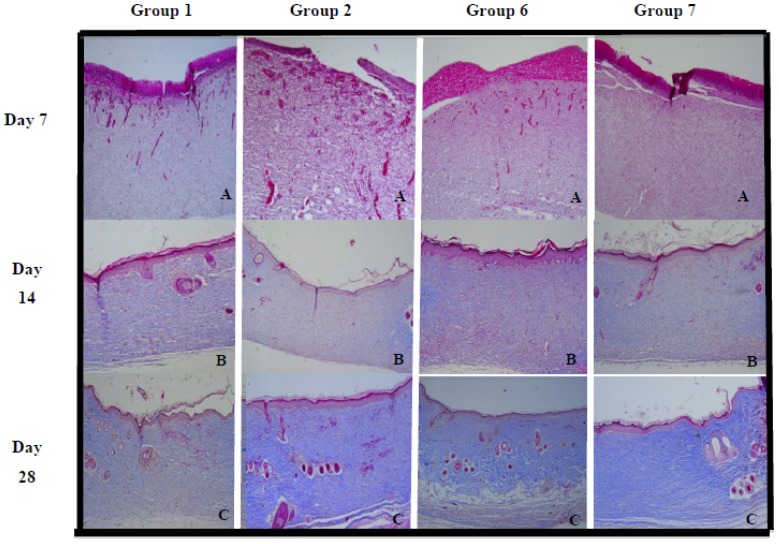
Evidence of collagen deposition on wound for animals treated with mahanimbicine (G2) and extract (G6) along with wound treated with standard ointment (G7) and untreated wound (G1).

Absence of collagen on the area beneath the wound was noticed as there was only inflammatory cells on the first week of excision (A, Figure 3). Movements of collagen in the region underneath the wound begin to appear on the 14th day post-wounding and complete re-structuring of collagen was significantly seen on the 28th day, especially in wounds treated with extract and mahanimbicine (**2**) (B, Figure 3) compared to rest of the treatment groups. Presence of blue bands beneath the epidermal layer indicated the presence of collagen deposition. Deposition of collagen up to 91.78% was seen in wounds treated with extract, while wounds treated with mahanimbicine (**2**) reached 88.54% (C, Figure 3). Table 3 shows the density of collagen based on the day interval.

Wound healing is a sequel of complex natural regeneration processes of skin cells to minimize or to eliminate scarring as well as to help healing and renewal of damaged cells. The common process of cutaneous healing occurs in three overlapping phases of coagulation and inflammation, proliferation and remodeling [3]. The processes of coagulation and inflammation begin immediately after injury, followed by platelet aggregation to control excessive blood loss from damaged blood vessels causing an influx of white blood cells into the wound site and mediation of multiple wound healing actions such as the release of protease for wound debridement, phagocytosis of debris and bacteria besides secretion of various and growth factors which leads to migration and division of cells involved in proliferative phase [21,22]. During this phase, angiogenesis, collagen deposition, epithelialization and newly formed granulation tissue consisting of endothelial cells, macrophages, fibroblasts and the components of new provisional extracellular matrix begin to cover and fill the wound area [22]. The remodeling phase involves collagen cross-linking and re-organization, evolution of granulation tissue into the scar tissue and cells that no longer needed are removed via apoptosis.

**Table 3:** Effect of carbazole alkaloids [mahanine (G2), mahanimbicine (G3), mahanimbine (G4)), essential oil (G5) and extract (G6) of *Murraya koenigii*, normal healing (G1) and standard ointment (G7) on percentage of collagen deposition on excision wound model in rats.

Group	Day 7 (%)	Day 14 (%)	Day 21 (%)	Day 28 (%)
1	22 ± 0.96	26.77 ± 1.27	61.84 ± 0.94	78.06 ± 1.22
2	20 ± 0.62	36.2 ± 1.36	65.63 ± 0.87	81.56 ± 1.04
3	20.6 ± 0.34	39.62 ± 0.29	67.76 ± 0.85	88.54 ± 1.34
4	14.87 ± 0.44	38.18 ± 0.69	67.15 ± 2.12	81.08 ± 1.09
5	19.18 ± 0.54	30.32 ± 2.33	63.23 ± 0.76	83.78 ± 1.24
6	19.75 ± 0.34	35.51 ± 1.44	69.41 ± 0.24	91.78 ± 1.02
7	21.08 ± 0.53	27.08 ± 1.03	60.05 ± 0.72	86.21 ± 1.12

n = 3 female Sprague-dawley rats per group, tabular value represent mean ± S.D, *p* ≤ 0.05.

Since *M. koenigii* has been used as folk medicine for many decades, and it is widely consumed as a part of spices in many Asian cuisines. Various scientific evidences have revealed the potential of *M. koenigii* in reducing diabetic mellitus, obesity and even as a chemopreventive agent [23,24]. In this present study, we investigated the potential of three carbazole alkaloids [mahanine (**1**), mahanimbine (**2**), mahanimbicine (**3**)], essential oil and extract of *M. koenigii* as a topical application in enhancing the wound healing process. Our findings revealed that the extract of *M. koenigii* significantly accelerated the rate of wound repair and granulation of new tissue by enhancing formation of more collagen, fibroblasts and hair follicles and by reducing the numbers of inflammatory cells. The surge in numbers of neutrophils and lympocytes elicited a response in inflammatory cells by releasing histamine, bradykinin and other factors that are essential to wound re-modelling and formation of scabs was observed as early as the 5th day post wounding as inflammation subsided with rapid wound contraction and wound debridement takes place. 

The healing process depends to a large extent on the regulated biosynthesis and deposition of new collagens and their subsequent maturation. Based on histological findings, extract of *M. koenigii* was found to enhance the formation of collagen significantly, followed by mahanimbicine (**2**). Collagen is the main structural protein component of connective tissue. Studies have reported that the collagen sponge enhances the formation of connective tissue and increases the vascularization of wounded tissues. Immediately upon injury, basal keratinocytes move from the basement membrane and interact with new connective tissue proteins in the dermis and wound bed inducing the expression of collagenase. Collagenase, a member of the matrix metalloproteinase family enzymes is responsible for degrading triple-helical fibrillar collagens into fragments, altering its structure and the affinities to which cells it binds [25,26]. Therefore, collagenase serves a beneficial role in wound healing during re-epithelialization by facilitating the movement of keratinocytes over the collagen rich dermis.

Although the epithelialization and contraction process had taken place on the surface within two weeks, the movement of fibroblasts in the wound area facilitates matrix formation and collagen is laid down over and throughout the amorphous “plastic tissue”. Movement of collagen was observed in all the groups on the 14th day post wounding where formation of new hair follicle buds and granulation of new skin tissue appears. On the 21st day post wounding, appearance of keratinocytes were very visible and more budding of new hair follicles and sebaceous glands were observed. By the 28th day, a near complete construction of dermal tissue was detected in the wounds treated with extract of *M. koenigii*, followed by wounds treated with mahanimbicine (**2**) compared to the wounds of the other experimental groups.

The healing potential of *M. koenigii* could be a result of the anti-microbial [27,28] and antioxidant properties exhibited by this perennial plant [29,30]. When the skin is open to infections, the normal healing process is disrupted as the inflammatory phase becomes chronic and suppresses the proliferation phase of healing, leading to delays in wound closure. The suppression of the production of free radicals at or around the wound bed caused by the anti-oxidant potential of this herb helps in reducing inflammation, increasing angiogenesis and collagen deposition. In previous studies, we had reported the selective antibacterial activities of these carbazole alkaloids and essential oil of *M. koenigii* against several clinical human pathogens [31]. We found that certain functional moieties play a vital role in influencing the bioactivity of these carbazole alkaloids, although they all share a similar skeleton. This can be correlated with bacteriostatic activity with its scavenging action on superoxide and hydroxyl radicals. In this present investigation, it is very clear that the synergistic effect of these carbazole alkaloids together with other phytochemicals found in *M. koenigii* extract could be responsible for reducing inflammation through antibacterial protection of wounds and this leads to increased angiogenesis and collagen deposition.

## 3. Experimental

### 3.1. Plant Material

Pest free leaves of *Murraya koenigii* (L.) Spreng were collected from Kg. Bobot, Kota Belud, Sabah in August 2010. Voucher specimens were deposited at the BORNEENSIS, Herbarium of the Institute for Tropical Biology and Conservation, Universiti Malaysia Sabah (BORH 37581).

### 3.2. Preparation of Extract and Isolation of Carbazole Alkaloids

Air-dried *M. koenigii* leaves (270 g) were extracted with ethanol (3 L, Merck, Darmstadt, Germany) for 7 days using a Soxhlet apparatus. Extracts were concentrated under reduced pressure at 40 °C to yield a dark green residue (14.75 g). A portion (5 g) was then subjected to successive silica gel (Kiesegel 60, 70–230 mesh, Merck) column chromatography with a stepwise gradient of hexane/ethyl acetate (Hex/EtOAc: 9:1, 8:2,7:3, 6:4, 1:1) before the column was washed with chloroform-methanol-water (65:25:4) to obtain six fractions of different polarity. Fractions were then profiled using a high performance liquid chromatography (HPLC) system equipped with a UV-Vis detector monitored at 254 nm (Prominence, Shimadzu, Kyoto, Japan) coupled with a reverse phase Phenomenex C-18 ODS (10 mm × 250 mm × 5 μm) column. Mobile phase in gradient elution was: A: 50% MeCN: 50% H_2_O, B: 100% MeCN with the profile of: 0–30 min: A: 30% B: 70%, 30.01–60.00 min A: 0% B: 100% and flow rate was set at 2.00 mL/min under 40 °C. A manual injection of 50 μL of pre-filtered solution (0.2 μm nylon membrane syringe filters, Whatman, New York, NY, USA) was analyzed. A total of three major alkaloids were isolated from *M. koenigii*, compound **1** (0.40%) from fraction 3 while compounds **2** (0.24%) and **3** (0.66%) from fraction 2.

Isolated peaks were subjected to Thin Layer Chromatography (TLC) confirmation with Drangendoff spray and ^1^H-NMR revealed the purity of the isolates and confirmed the identity of the alkaloids with the presence of aromatic and amine protons. Three compounds were isolated and subjected to ^1^H-, ^13^C- and 2D NMR spectroscopic analyses. The structures of compounds were determined based on the comparison of their ^1^H- and ^13^C-NMR data with those reported in the literature. All spectral data were obtained on the following instruments: IR on a ThermoNicolet FT-IR spectrometer, optical rotations was measured on an AUTOPOL IV automatic polarimeter (Rudolph Research Analytical), ^1^H-NMR (600 MHz) and ^13^C-NMR (150 MHz) were recorded with a JEOL ECA 600 (Japan Electronic Optics Laboratory Co. Ltd., Tokyo, Japan) spectrometer, with TMS as internal standard. HR-ESI-TOFMS data were obtained using LCMS-IT-TOF (Shimadzu, Tokyo, Japan).

The isolated compounds were subjected to spectroscopic measurements and based on independent structure elucidation the structures of these three compounds were determined as mahanine (**1**) (C_23_H_25_NO_2_), mahanimbicine (**2**) (C_23_H_25_NO) and mahanimbine (**3**) (C_23_H_25_NO) (Figure 1). The spectroscopic data of the respective compounds are assigned as follows: 

*Compound*** 1**; C_23_H_25_NO_2_; white powder; [a]_D_^25^ = +9.0° (CHCl_3_, *c* 2.0):^1^H NMR: δ 1.41 (3H, s, 3-CH_3_), 1.56 (3H, br s, 4′-CH_3_), 1.64(3H, br s, 4′′-CH_3_), 1.72 (2H, m, H-1′), 2.17(2H, m, H-2′), 2.27 (3H, br s, 5-CH_3_), 5.01 (1H, br s, OH), 5.12 (1H, m, H-3′), 5.64(1H, d, *J* = 9.8 Hz, H-2), 6.82 (1H, d, *J* = 9.8 Hz, H-1), 6.62 (1H, d, H-8), 6.81 (1H, d, *J* = 2.2 Hz, H-10), 7.47 (1H, s, H-6), 7.64(1H, d, *J* = 8.3 Hz, H-7), 7.65 (1H, br s, NH), ^13^C NMR: δ 16.4 (5-CH_3_), 17.8 (4′-CH_3_), 24.2 (2′), 26.7 (3-CH_3_), 26.5 (4′′-CH_3_), 42.2 (1′), 79.2 (3), 97.9 (10), 105.8 (11b), 109.1 (8), 118.09 (6a), 119.6 (1), 118.1 (7a), 118.5 (5), 120.6 (7), 121.1 (6), 125.7 (3′), 129.2 (2), 132.2 (4′), 136.9 (11a), 143.3 (10a), 148.9 (4), 156.5 (9). EIMS 70 eV *m/z* (mass value): 347 [M]^+^ (100), 332 (19), 304 (8), 278 (15), 264 (75).

*Compound*** 2**; C_23_H_25_NO; mild green powder; [a]_D_^25^ = +60.0° (CHCl_3_, *c* 0.30):^1^H-NMR:δ1.41 (3H, s, 3-CH_3_), 1.64 (3H, s, 4′-CH_3_), 1.57 (3H, s, 4′′-CH_3_), 1.72 (2H, m, H-1′), 2.17 (2H, m, H-2′), 2.46 (3H, s, 8-CH_3_), 5.12 (1H, m, H-3′), 5.68 (1H, *J* = 9.8 Hz, H-2), 6.85 (1H, d, *J* = 9.8 Hz, H-1), 6.57 (1H, d, *J* = 8.5Hz, H-5), 7.09 (1H, d, *J* = 1.7, 8.3 Hz, H-9), 7.27(1H, br d, *J* = 8.3 Hz, H-10), 7.69 (1H, d, *J* = 1.7 Hz, H-7), 7.71 (1H, d, *J* = 8.5Hz, H-6), 7.78 (1H, s, NH).^13^C-NMR: δ 17.8 (4′′-CH_3_), 21.7 (8-CH_3_), 24.0 (C-2′), 25.7 (4′-CH_3_), 26.6 (3-CH_3_), 42.3 (C-1′), 79.6 (C-3), 106.0 (C-11b), 109.7 (C-5), 111.5 (C-10), 119.3 (C-1), 118.8 (C-6a), 120.1 (C-7), 121.2 (C-6), 125.6 (C-3′), 125.3 (C-7a), 126.7 (C-9), 129.3 (C-2), 129.4 (C-8), 132.6 (C-4′), 138.7 (C-11a), 140.2 (C-10a), 152.9 (C-4). EIMS 70 eV *m/z* (mass value): 331[M]^+^ (23), 316 (6), 248 (100), 210 (3).

*Compound*** 3**; C_23_H_25_NO; mild brownish-yellow powder; [a]_D_^25^ = +40.0° (CHCl_3_, *c* 0.80): ^1^H-NMR: δ 1.41(3H, s, 3-CH_3_), 1.63 (3H, s, 4′-CH_3_), 1.55 (3H, s, 4′′-CH_3_), 1.72 (2H, t, H-1′), 2.17 (2H, m, H-2′), 2.29 (3H, s, 5-CH_3_), 5.11 (1H, m, H-3′), 5.63 (1H, d, *J* = 9.8 Hz, H-2), 6.84 (1H, d, *J* = 9.8 Hz, H-1), 7.23 (1H, t, *J* = 8.1 Hz, H-8), 7.07 (1H, br t, *J* = 8.1 Hz, H-9), 7.85 (1H, br d, *J* = 8.1 Hz, H-10), 7.61 (1H, s, H-6), 7.85 (1H, br s, NH), 7.36(1H, d, *J* = 7.8 Hz, H-7).^13^C NMR: δ 16.2(5-CH_3_), 17.6 (4′′-CH_3_), 23.9 (C-2′), 25.8 (4′-CH_3_), 26.4(3-CH_3_), 42.1 (C-1′), 79.3 (C-3), 105.5 (C-11b), 119.9 (C-10), 24.8 (C-6a), 119.3 (C-1), 118.3 (C-5), 111.4 (C-7), 124.9 (C-8), 121.8 (C-6), 128.9 (C-2), 141.6 (C-7a), 125.5 (C-3′), 119.7 (C-9), 132.3 (C-4′), 136.9 (C-11a), 117.9 (C-10a), 150.9 (C-4). EIMS 70 eV *m/z* (mass value): 331[M]^+^ (24), 248 (100), 210 (5). 

### 3.3. Extraction of Essential Oil and Analysis

Fresh leaves of *M. koenigii* (50 g) were chopped and subjected to hydro-distillation using a Clevenger-type apparatus for duration of 8 h. Distilled oil were collected in pentane, dried over anhydrous Na_2_SO_4_, concentrated *in vacuo*, stored in air-tight glass vials flushed with nitrogen (N_2_) gas and kept at −20 °C prior to analysis. Analysis of the essential oil was performed using a Shimadzu QP-2010 gas chromatograph coupled with a Shimadzu GCMSQP-2010Plus detector (Shimadzu, Japan) using a SGE BPX-5 (30.0 m × 0.25 μm i.d., film thickness 0.25 μm) fused silica capillary column. High purity helium was used as the carrier gas at a constant flow rate of 0.8 mL/min. A total of 1 μL sample was injected (split ratio 100:1) into GCMS using AOC5000 auto injector for analysis. The initial temperature was set at 50 °C, heated at a rate of 3 °C/min to 280 °C and held isothermally for 5 min. Ion source temperature for these analysis was set at 200 °C while the interface temperature was set at 280 °C and the mass spectrometer was set to operate in electron ionization mode with an ionizing energy of 70 eV as acquisition mass range from 40 a.m.u to 450 a.m.u. at 0.25 scan/s.

Identification of volatile organic constituents was confirmed using published electron impact-mass spectra (EI-MS) in the National Institute for Standard and Technology (NIST) 1998 and Shimadzu’s Flavours and Fragrance of Natural and Synthetic Compounds (FFNSC) version 1.2 computerized mass spectral libraries. The retention indices were determined based on a homologous series of *n*-alkanes (C_8_–C_40_; Custom Retention Time Index Standard, Restek Corp, New York, NY, USA) external standard analyzed under the same operating conditions and calibrated based on the Automatic Adjustment of Compound Retention Time (AART) function of the GCMS. Relative concentrations of the essential oil components were calculated based on GC peak area with the AART correction factors.

### 3.4. Wound Healing Activity

#### 3.4.1. Experimental Animals

Healthy female Sprague Dawley rats weighing between 200–250 g were obtained from the Animal Laboratory of Universiti Sains Malaysia, Kubang Kerian, Kelantan. The Institutional Animal Ethics Committee approved this animal experimentation study at Universiti Malaysia Terengganu (UMT), Terengganu in accordance with OECD guidelines (No.404, OECD, 2004) together with current guidelines for the care of laboratory animals. All animals were housed in standard environmental conditions with temperature of 25 ± 1 °C with 12 h light and 12 h of dark cycle and were acclimatized to hygienic laboratory condition for a period of 7 days before the experiment was carried out. Any change in clinical signs such as diarrhea, food and water intake, behavior and blood in urine was observed. Animals were fed with standard commercial pellet diet (10% of their body weight) and distilled water *ad libitum*. 

#### 3.4.2. Grouping of Animals

A total of 84 rats were divided into seven experimental groups of twelve rats for four time intervals (day 7, 14, 21, 28): Group I (*negative control group*): animals in this group did not receive administration of any wound healing agent; Group II: animals in this group received administration of mahanine (**1**); Group III: animals in this group received administration of mahanimbicine (**2**); Group IV: animals in this group received administration of mahanimbine (**3**); Group V: animals in this group received administration of essential oil of *M. koenigii*; Group VI: animals in this group received administration of *M. koenigii* extract.; Group VII (*positive control group*): animals in this group received the administration of a commercial wound healing cream, Chlorocresol BP 0.1% (Sunward Pharma, Selangor, Malaysia) as reference drug.

#### 3.4.3. Excision Wound Model and Treatment of Wounds

Animals in assigned groups were anaesthetized by the open mask method with anesthetic ether before wound creation. Hair on the dorsal thoracic region of the animal was trimmed using electric clippers, then depilated and treated with a swab of 70% alcohol as topical disinfection before performing the wound creation. A wound of 8 mm diameter was excised by using sharp sterile skin bio-puncher (FRAY^TM^, New York, NY, USA). Wounds was created on clean shaved skin by a uniformly pressure single twist until the subcutaneous dermal layers were separated. Each animal was wounded with two circular full thickness wounds on the dorsal part individually to represent duplicates. Hemostasis was achieved by even compression on the wound with sterile gauze. Animals were housed individually to prevent external tampering with the wounds. The tested carbazole alkaloids, essential oil and extract (50 mg) were prepared by incorporating into an ointment base (vehicle) consisting of blank placebo (Sunward Pharma). Topical application of a thin layer of carbazole alkaloids, essential oil and extract of *M. koenigii* ointment was done twice daily at the same time for a period of 14 days.

#### 3.4.4. Wound Contraction and Epithelialization Time

The excision wound margin was traced after wound creation by using transparent paper and the areas were measured using graph paper. Wound contraction (w.c) was measured daily until the wound healed completely and expressed as the percentage of reduction in wound area (w.a) of the original surgical excision as follows:

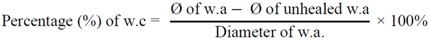



The epithelialization times were measured from the initial day the excision was performed.

#### 3.4.5. Histopathological Evaluation

Cross-sections of skin, kidney and liver specimens from each group were collected on days 7, 14, 21 and 28 of the experiment for histopathological evaluation and determination of any cytotoxic effects on vital organs. All specimens were fixed in 10% buffered formalin, dehydrated through a graded alcohol series, cleared in xylene and blocked with paraffin before sectioning into 5 μm sections. Serials of 5 μm sections of skin, kidney and liver were stained with Hematoxylin and Eosin (H&E) for cytotoxicity evaluation and additional sections of skin were stained specifically with Masson trichrome for the assessment of collagen content and maturation within the dermis. The sections were then examined under light microscope, observed from the aspects of fibroblast proliferation, collagen formation, angiogenesis, epithelialization, necrosis of liver and kidney cells and photomicrographs were taken.

#### 3.4.6. Computerized Collagen Density Evaluation

Skin sections stained with Masson trichrome were examined under polarized light microscope (Leica, Hamburg, Germany) using image analyzer software (Leica application suite ver. 4.0) and measurements were made of the intensity of the blue colour that indicates collagen deposition. Density of collagen deposition under the wound area were measured and compared to normal dermis collagen at 100´ magnification. Mean values of collagen from normal dermis were accepted to be equivalent to 100. Mean values of collagen density under wound areas were expressed as a percentage ratio compared to collagen density of normal dermis during post wounding days:

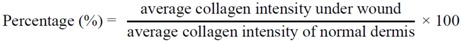



### 3.5. Statistical Analysis

The results were analyzed statistically using Student’s *t*-test to identify differences between treated and control. The data were considered significant at *p* < 0.05. 

## 4. Conclusions

Absence of necrotic cells, lesion or shrinkage in cells of liver and kidney of the animals were observed, suggesting the non-toxic nature of the treatments using extract, mahanine (**1**), mahanimbicine (**2**), mahanimbine (**3**) and essential oil of *M. koenigii*. Therefore, the use of *M. koenigii* extract as wound healing agent could be useful as it protects the injury site from infections and rapidly increases the rate of connective tissue formation.
